# William Henry Eames, D. D. S.

**Published:** 1894-05

**Authors:** A. H. Fuller


### Obituary.

### WILLIAM HENRY EAMES, D.D.S.

Dr. William Henry Eames died in St. Louis, at the residence of his daughter, Dr. Emma Eames Chase, on March 29, after a short illness.

He was born in Auburn, N. Y., August 23, 1828. His father, Georges Eames, soon after the birth of this son, removed to Oneida County, N. Y., where his father, James Eames, with his large family had settled in 1794. They were progressive people, taking an active part in all public affairs, and particularly interested in educational advancement. The active interest, untiring zeal, and devotion with which Dr. Eames labored for the advancement of his profession, in the college, in the journals, and in societies, was a direct continuation of the spirit with which his father and grandfather before him had worked for the intellectual advancement of the rising generation. He was educated in the public schools, and at the Clinton Academy, in Oneida County. After teaching school several years he commenced the study of medicine with Dr. Porter, an eminent English physician, and went to Ann Arbor to complete his medical studies at the Michigan University. While there he decided to study dentistry, and entered the Ohio Dental College, graduating in 1853. He returned to Ann Arbor and formed a partnership with his preceptor, Dr. Porter. He was married in 1855 to Laura M. Scofield, of Clinton, Mich., and removed to Lebanon, Tenn., in 1857, remaining there until 1862, when, owing to the disturbed condition of the South, he removed to St. Louis, Mo. He at once became a member of the St. Louis Dental Society, and always took an active and prominent part in the profession in St. Louis. It was at a meeting of the Society at his residence that the subject of organizing a dental college in St. Louis was introduced. There were present at this meeting Drs. Judd, McKellops, Forbes, Peebles, and others, and from its first inception he was an active worker, continuing through many years to the very night before he was stricken down. He was one of the founders of the *Missouri Dental Journal*, and for many years its editor, and later, editor of the *Archives of Dentistry*, which succeeded it. Dr. Eames was an active member of the American Dental Association, the Missouri State Dental Association, and the St. Louis Dental Society; the last two of which he has been president of at different times. He had also served as president of the Association of Dental College Faculties, and was an honorary member of the Illinois, Iowa, and other State and district dental societies. It is not necessary to speak here of the genial nature and the many admirable qualities of our departed brother; these are known to all who ever had the pleasure of his acquaintance, and will be referred to as pleasant recollections until all have followed him to the other side.

The doctor leaves a wife and seven grown children,—two sons and five daughters.

A. H. Fuller.

